# Efficacy of irreversible EGFR-TKIs for the uncommon secondary resistant *EGFR* mutations L747S, D761Y, and T854A

**DOI:** 10.1186/s12885-017-3263-z

**Published:** 2017-04-19

**Authors:** Masato Chiba, Yosuke Togashi, Eri Bannno, Yoshihisa Kobayashi, Yu Nakamura, Hidetoshi Hayashi, Masato Terashima, Marco A. De Velasco, Kazuko Sakai, Yoshihiko Fujita, Tetsuya Mitsudomi, Kazuto Nishio

**Affiliations:** 1 Kindai University Faculty of Medicine, 377-2 Ohno-higashi, Osaka-Sayama, Osaka, 589-8511 Japan; 2 Thoracic Surgery, Kindai University Faculty of Medicine, Osaka, Japan; 3 Medical Oncology, Kindai University Faculty of Medicine, Osaka, Japan

**Keywords:** *EGFR* mutation, Secondary resistant mutation, L747S, D761Y, T854A, Irreversible EGFR-TKI

## Abstract

**Background:**

Non-small cell lung cancer (NSCLC) harboring common epidermal growth factor receptor (EGFR) gene mutations (exon 19 deletion or exon 21 L858R) respond to EGFR tyrosine kinase inhibitors (EGFR-TKIs). The secondary T790 M mutation in exon 20 of the *EGFR* gene is the most common type of acquired resistance mutation. Several reports have also shown that other secondary mutations (L747S, D761Y and T854A), while uncommon, can induce acquired resistance to first-generation EGFR-TKIs. However, little is known about the anticancer activities of second- or third-generation EGFR-TKIs.

**Methods:**

Uncommon secondary mutations were introduced into Ba/F3 cells along with the sensitive *EGFR* L858R mutation (Ba/F3-L858R/L747S, Ba/F3-L858R/D761Y, and Ba/F3-L858R/T854A), and the sensitivities to various EGFR-TKIs were then investigated.

**Results:**

Both the Ba/F3-L858R/L747S and Ba/F3-L858R/D761Y cell lines exhibited weak resistances to first-generation reversible EGFR-TKIs, while the Ba/F3-L858R/T854A cell line exhibited a strong resistance. In contrast, irreversible EGFR-TKIs, especially third-generation EGFR-TKIs, were capable of overcoming these resistances. Western blot analyses demonstrated that gefitinib (first-generation) inhibited the phosphorylation of EGFR to a lesser extent in cells with these secondary mutations than in cells with the sensitive L858R mutation alone. In contrast, afatinib and osimertinib (second- and third-generation) inhibited the phosphorylation of EGFR in cells with these secondary mutations to a similar extent as that seen in cells with the sensitive L858R mutation alone.

**Conclusions:**

Our experimental findings suggest that irreversible EGFR-TKIs, especially third-generation EGFR-TKIs, can be effective against uncommon secondary mutations and that switching to third-generation EGFR-TKIs could be a promising treatment strategy for patients with acquired resistance because of these uncommon secondary mutations.

## Background

Lung cancer is the leading cause of cancer-related mortality worldwide [1, 2]. The epidermal growth factor receptor (EGFR) is recognized as an important molecular target in cancer therapy, and somatic activating mutations of the *EGFR* gene (*EGFR* mutations) are known as one of the oncogenic driver mutations in non small cell lung cancer (NSCLC). NSCLCs with *EGFR* mutations are associated with sensitivity to EGFR tyrosine kinase inhibitors (EGFR-TKIs) [3].

Gefitinib and erlotinib are first-generation (1G) reversible EGFR-TKIs that are highly effective against NSCLC carrying common activating *EGFR* mutations (exon 19 deletion or exon 21 L858R) [4–8]. Although most patients respond dramatically to such treatments, the majority eventually experience disease progression [9]. Many studies have revealed several resistance mechanisms and candidates, including the secondary *EGFR* mutation T790 M [10] and other uncommon mutations (L747S [11], D761Y [12], and T854A [13]), *MET* amplification [14], *HER2* amplification [15], *PTEN* down-regulation [16], high-level HGF expression [17], epithelial-mesenchymal transition [18], and conversion to small cell lung cancer [19] (for review, see [20, 21])**.**

Afatinib, a second-generation (2G) irreversible EGFR-TKI, also exhibits a marked efficacy against NSCLC carrying *EGFR* mutations, similar to the effects of gefitinib and erlotinib [19, 22]. In addition, afatinib can be effective against uncommon *EGFR* mutations [23, 24] for which 1G–TKIs are less effective [25, 26]. Apparently, not all *EGFR* mutations are created equal; thus, different *EGFR* mutations may have different sensitivities to various EGFR-TKIs [27].

The secondary T790 M mutation in exon 20 of the *EGFR* gene is the most common type of acquired resistance mutation. Approximately 50% of cases with acquired resistance to EGFR-TKI therapy carry this T790 M mutation in the kinase domain of *EGFR* as well as an *EGFR*-activating mutation [28–30]. Several recent studies have demonstrated that third-generation (3G) irreversible EGFR-TKIs, which are mutant-selective inhibitors, can overcome T790 M-mediated resistance [31–34]. These findings suggest that different *EGFR* mutations have different sensitivities to EGFR-TKIs.

Although uncommon, there have been several reports showing that other secondary mutations (L747S [11], D761Y [12], and T854A [13]) induce resistance to 1G–TKIs. The anticancer activities of 2G- or 3G–TKIs against these uncommon secondary mutations, however, remain unclear. In the present study, the anticancer activities of various EGFR-TKIs (1G, 2G, or 3G) against uncommon secondary *EGFR* mutations were investigated in vitro using the murine Ba/F3 cell system. The Ba/F3 cell system is a murine pro-B cell line that is dependent on interleukin-3 (IL-3) for its survival and growth and is a well-validated and widely used cell system. The ability of Ba/F3 cells transfected with a mutated version of the gene to proliferate in the absence of IL-3 indicates an oncogenic ability [35, 36].

## Methods

### Cell cultures and reagents

**Fig. 1:**
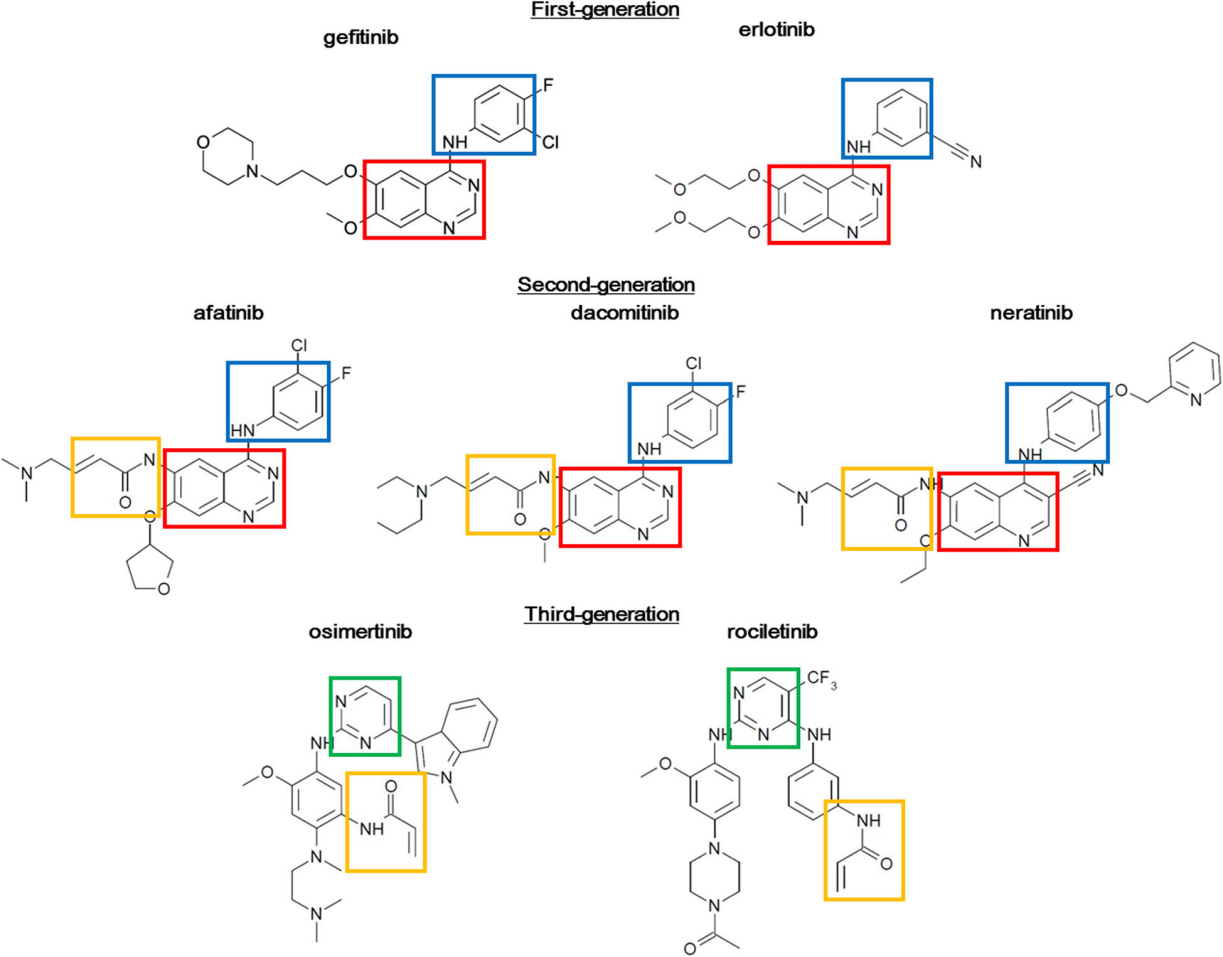
Structures of EGFR-TKIs used in this study. The first- and second-generation EGFR-TKIs both have anilino (*blue square*)-quinazoline (*red square*) structures. However, the second-generation TKIs also have an acrylamide group (*orange square*), which serves as a chemically reactive Michael acceptor electrophile that targets a cysteine nucleophile (Cys797), resulting in a covalent adduct. The third-generation EGFR-TKIs are pyrimidine (*green square*)-based compounds with an acrylamide group (*orange square*) for covalent binding to the EGFR

The murine pro-B cell line Ba/F3 (RCB0805) was provided by the RIKEN Bio Resource Center (Tsukuba, Japan). Ba/F3 cells were maintained in Roswell Park Memorial Institute (RPMI) 1640 medium (Sigma-Aldrich, St. Louis, MO)**,** supplemented with 10% fetal bovine serum (FBS) (GIBCO BRL, Grand Island, NY) and 10 ng/mL of IL-3 (Cell Signaling Technology) in a humidified atmosphere of 5% CO_2_ at 37 °C. Gefitinib and erlotinib (1G–TKIs), afatinib, dacomitinib, and neratinib (2G–TKIs), and osimertinib and rociletinib (3G–TKIs) were purchased from Selleck Chemicals (Houston, TX). The structures of these agents are summarized in Fig. 1.

### Protein crystal structure

The crystal structure of EGFR was drawn using the PyMOL Molecular Graphics System (Version 1.7.4; Schrodinger, LLC) based on crystal structure information from PDB ID 2ITZ (*EGFR* L858R mutation in complex with gefitinib), as previously described [24].

### Database analysis

To analyze the prevalence of *EGFR* L747S, D761Y, and T854A mutations, the Cancer Genome Atlas (TCGA) dataset (http://cancergenome.nih.gov/) [37, 38] and the Catalogue of Somatic Mutations in Cancer (COSMIC) database (http://cancer.sanger.ac.uk/cancergenome/projects/cosmic/) were used.

### Plasmid construction, viral production and stable transfectants

The methods used in the present study have been previously described [24]. Briefly, pBABE with a full-length wild-type *EGFR* cDNA fragment was purchased from Addgene (Cambridge, MA). pBABE constructs encoding the *EGFR* L858R mutation and the *EGFR* L858R mutation plus each of the resistant mutations (L858R + L747S, L858R + D761Y, L858R + T854A, and L858R + T790 M) were generated using the PrimeSTAR Mutagenesis Basal Kit (TaKaRa, Otsu, Japan). All primer sequences are available upon request. All the mutations were confirmed using direct sequencing experiments. The pBABE constructs were cotransfected with a pVSV-G vector (Clontech, Mountain View, CA) to generate the viral envelope in gpIRES-293 cells using the FuGENE6 transfection reagent (Roche Diagnostics, Basel, Switzerland) to produce viral particles. After 48 h of transfection, the culture medium was collected and the viral particles were concentrated by centrifugation at 15,000 ×*g* for 3 h at 4 °C. The viral pellet was then resuspended in Dulbecco’s Modified Eagle’s Medium (DMEM) (Sigma-Aldrich, St. Louis, MO) and was added to Ba/F3 cells. Infected Ba/F3cells were then purified using GFP**-**based fluorescence-activated cell sorting using the BD FACS Aria Cell Sorter Special Order Research Product (BD Biosciences, Franklin Lakes, NJ).

### Antibodies

Rabbit antibodies specific for EGFR, phospho-EGFR, and β-actin were obtained from Cell Signaling (Beverly, MA).

### Western blot analysis

Western blot analysis was performed as previously described [39]. Briefly, Transfected Baf/3 cells were cultured to subconfluence and were rinsed with phosphate-buffered saline (PBS) and harvested with Lysis A buffer containing 1% Triton X-100, 20 mM Tris-HCl (pH 7.0), 5 mM EDTA, 50 mM sodium chloride, 10 mM sodium pyrophosphate, 50 mM sodium fluoride, 1 mM sodium orthovanadate, and a protease inhibitor mix (Complete™; Roche Diagnostics). The total -cell lysate was subjected to SDS-PAGE and was blotted onto a polyvinylidene difluoride membranes. After blocking with 2.5% nonfat milk and 3% bovine serum albumin in a TBS buffer (pH 8.0) with 0.1% Tween-20, the membrane was probed with the primary antibody. After rinsing twice with TBS buffer, the membrane was incubated in primary and secondary antibodies, followed by visualization using an enhance chemiluminescence detection system and LAS-4000 (GE Healthcare, Buckinghamshire, UK). When the phosphorylation levels of EGFR and apoptosis-related molecules were investigated after inhibitor exposure, the samples were collected 3 and 8 h after stimulation, respectively.

### IL-3 independent cell growth assay

The transfected Ba/F3 cell lines were cultured for 72 h without IL-3 and were then analyzed using a 3,4,5-dimethyl-2H-tetrazolium bromide assay (MTT; Sigma-Aldrich, St. Louis, MO). The experiment was performed in triplicate as previously described [24].

### Growth inhibition assay in vitro

The growth-inhibitory effects of EGFR-TKIs were examined using an MTT assay [40]. When Ba/F3 transfectant cell lines were used, the cells were cultured without IL-3. Each experiment was performed in triplicate**,** as previously described [24].

### Statistical analysis

Continuous variables were analyzed using the Student *t*-test, and the results were expressed as the average and standard deviation (SD). The statistical analyses were two-tailed and were performed using Microsoft Excel (Microsoft, Redmond, WA). A *P* value of less than 0.05 was considered statistically significant.

## Results

### Crystal structure of EGFR, sites of L747, D761, and T854, and frequencies of these secondary mutations in the database

**Fig. 2:**
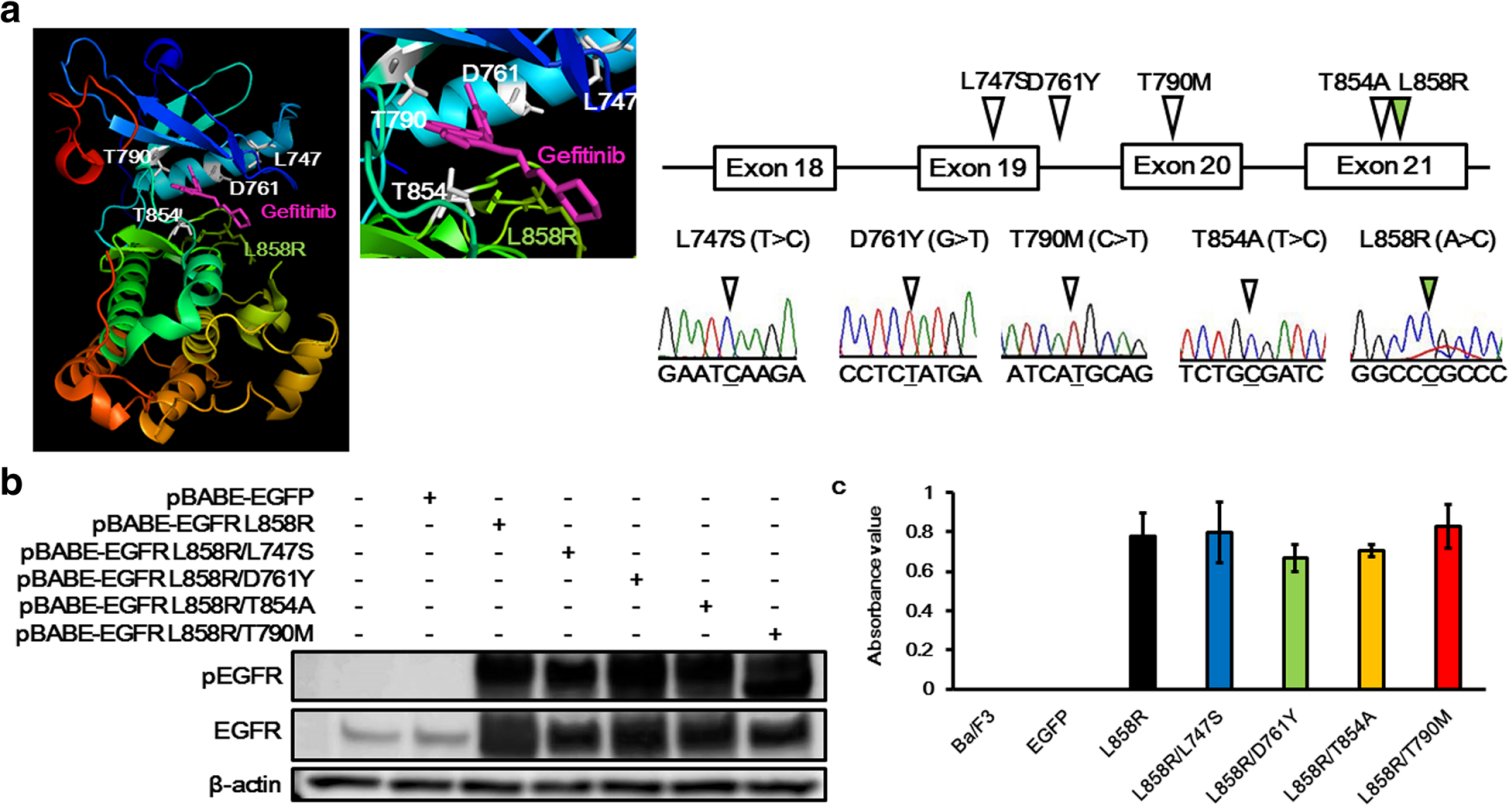
Ba/F3 cell lines harboring secondary mutations along with the *EGFR* L858R mutation exhibited IL-3-independent growth. **a** Crystal structure of EGFR. This figure was drawn using the PyMOL Molecular Graphics System based on crystal structure information from PDB ID 2ITZ (*EGFR* L858R mutation in complex with gefitinib). L747 is located at the start of the loop between the h3 strand and the α-C-helix, D761 is located in the α-C-helix, and T854 is located in the activation loop of EGFR. These residual positions (L747, D761, and T854) are close to the binding sites of ATP or reversible EGFR-TKIs. The secondary mutations were introduced into *EGFR* along with the L858R mutation. The mutations were confirmed using direct sequencing. **b** Expression of EGFR in the transfectant Ba/F3 cell lines. The expression of EGFR was confirmed using western blotting. The phosphorylation levels of EGFR were also elevated, similar to that in cells with the L858R mutation alone. β-actin was used as an internal control. **c** Ba/F3 assay. The cellular growth of Ba/F3 transfectant cell lines grown in the absence of IL-3 were evaluated using an MTT assay. The Ba/F3 and Ba/F3-EGFP cell lines could not grow without IL-3, while the other cell lines (L858R, L858R/L747S, L858R/D761Y, L858R/T854A, and L858R/T790 M) were able to grow without IL-3. Column, mean of independent triplicate experiments; error bars, SD

The crystal structure of EGFR was drawn using the PyMOL Molecular Graphics System based on crystal structure information from PDB ID 2ITZ (*EGFR* L858R mutation in complex with gefitinib). L747 is located at the start of the loop between the h3 strand and the α-C-helix, D761 is located in the α-C-helix, and T854 is located in the activation loop of EGFR. As shown in Fig. 2a, these residual positions (L747, D761, and T854) are close to the binding sites for ATP or reversible EGFR-TKIs (Fig. 2a).

In the TCGA dataset, a total of 408 NSCLC samples (230 adenocarcinomas and 178 squamous cell carcinomas) that had not been treated with chemotherapy, including EGFR-TKIs, were analyzed, and 30 samples had *EGFR* mutations in exons 18–21. One sample had the T790 M mutation coupled with L858R, but none of the other samples carried uncommon secondary mutations. In the COSMIC database, very few cases of L747 (32, 0.17%; P, 17; S, 14; V, 1), D761 (17, 0.092%; Y, 10; N, 5; G, 2), or T854 (7, 0.038%; A, 4; I, 1; P, 1; S, 1) substitution mutations were found among 18,315 *EGFR* mutations in exons 18–21. These findings suggest that the frequencies of L747, D761, and T854 substitution mutations are very low.

### All *EGFR* mutation (L858R/L747S, L858R/D761Y, or L858R/T854A)-derived Ba/F3 cell lines can grow without IL-3

To investigate the various EGFR-TKIs sensitivities of these uncommon secondary mutations, *EGFR*-overexpressed Ba/F3 cell lines were created and a Ba/F3 assay was performed. The *EGFR* L858R mutation was used as a sensitive mutation, and secondary mutations were introduced into the construct along with this L858R mutation (L858R/L747S, L858R/D761Y, L858R/T854A, and L858R/T790 M) (Fig. 2a). *EGFR*-overexpression was confirmed by western blotting in the transfectant Ba/F3 cell lines (Fig. 2b). All the Ba/F3 cell lines harboring these secondary mutations along with the L858R mutation exhibited IL-3-independent growth, similar to the Ba/F3-L858R cell line (Fig. 2c). The growth rates of the Ba/F3 cells transfected with each of the constructs were not significantly different, since the actual OD values at 72 h after seeding 2 × 10^3^ cells into each well were not significantly different (L858R, 2.23 ± 0.18; L858R/L747S, 2.73 ± 0.27; L858R/D761Y, 3.14 ± 0.21; L858R/T854A, 2.88 ± 0.06).

### Sensitivities to various EGFR-TKIs of transfectant Ba/F3 cell lines harboring secondary mutations

**Fig. 3:**
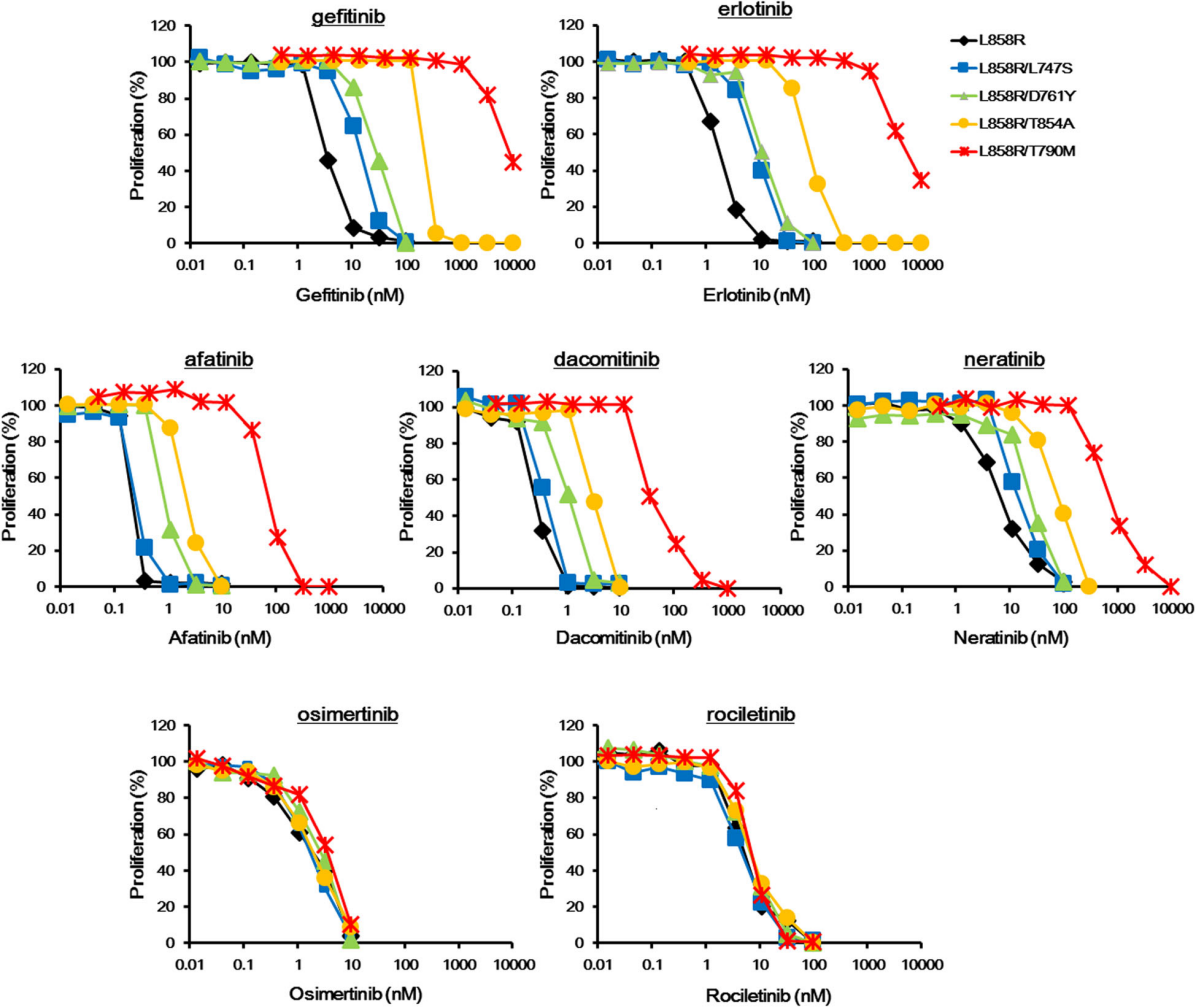
Growth inhibitory curves of the transfectant Ba/F3 cell lines. The growth inhibitory assay was performed using an MTT assay and was used to compare the sensitivities to various EGFR-TKIs (1G, gefitinib and erlotinib; 2G, afatinib, dacomitinib, and neratinib; 3G, osimertinib and rociletinib). The Ba/F3-L858R/L747S (*blue*) and Ba/F3-L858R/D761Y (*green*) cell lines were slightly resistant to 1G–TKIs (gefitinib and erlotinib), whereas the Ba/F3-L858R/T854A cell line (*orange*) was markedly resistant. In contrast, the degrees of resistance were weakened by the application of 2G–TKIs (afatinib, dacomitinib, and neratinib). Furthermore, 3G–TKIs were as effective against these Ba/F3 cell lines as they were against the Ba/F3-L858R cell line (*black*). In the Ba/F3-L858R/T790 M cell line (*red*), a similar tendency was observed. Lines, mean of independent triplicate experiments

A growth inhibitory assay was performed using an MTT assay, and the sensitivities of transfectant Ba/F3 cell lines to various EGFR-TKIs were compared. The growth inhibitory curves and the 50% inhibitory concentrations (IC_50_) are summarized in Fig. 3 and Table 1. The Ba/F3-L858R/L747S and Ba/F3-L858R/D761Y cell lines were slightly resistant to 1G–TKIs (gefitinib and erlotinib), whereas the Ba/F3-L858R/T854A cell line was markedly resistant. In contrast, the degrees of resistance were weakened by 2G–TKIs (afatinib, dacomitinib, and neratinib). Furthermore, 3G–TKIs (osimertinib, and rociletinib) were as effective against these Ba/F3 cell lines as they were against the Ba/F3-L858R cell line (Fig. 3 and Table 1). In the Ba/F3-L858R/T790 M cell line, a similar tendency was observed (Fig. 3 and Table 1).

**Table 1:** IC_50_ of various EGFR-TKIs in the transfectant Ba/F3 cell lines

EGFR-TKI		IC_50_ (nM)				
		L858R	L858R/L747S	L858R/D761Y	L858R/T854A	L858R/T790 M
First-generation	gefitinib	3.38 (1)	15.1 (5.6)	29.4 (8.7)	222 (68)	8568 (2535)
	erlotinib	1.82 (1)	8.68 (4.8)	11.1 (6.2)	85.9 (47)	5377 (2955)
Second-generation	afatinib	0.21 (1)	0.24 (1.1)	0.82 (3.9)	2.13 (10)	73.1 (116)
	dacomitinib	0.27 (1)	0.41 (1.5)	1.16 (4.3)	3.19 (12)	38.1 (146)
	neratinib	6.44 (1)	13.91 (2.2)	23.8 (3.7)	77.2 (12)	707 (110)
Third-generation	osimertinib	1.98 (1)	1.74 (0.9)	2.74 (1.4)	1.98 (1)	3.67 (1.3)
	rociletinib	5.22 (1)	4.69 (0.9)	6.71 (1.3)	6.94 (1.3)	7.08 (1.4)

*IC*_50_ 50% inhibitory concentration, *EGFR-TKI* epidermal growth factor receptor tyrosine kinase inhibitor

The numbers in parentheses indicate the percentages of the actual IC_50_ value for each mutant relative to that of L858R

### Comparison of IC_50_

To compare the sensitivities, the ratios of IC_50_ relative to that of the Ba/F3-L858R cell line (sensitive mutation) (IC_50_ ratios) were calculated, and these ratios are summarized in Table. 1. The IC_50_ ratios of the 1G–TKIs were around 5-fold in the Ba/F3-L858R/L747S and Ba/F3-L858R/D761Y cell lines, indicating that these secondary mutations induced a weak resistance to 1G–TKIs (Table 1). In contrast, the IC_50_ ratios of the 2G–TKIs were less than 5-fold and those of the 3G–TKIs were around 1-fold in the Ba/F3-L858R/L747S and Ba/F3-L858R/D761Y cell lines, indicating that these mutations were sensitive to irreversible EGFR-TKIs (Table 1). In Ba/F3-L858R/T854A, the IC_50_ ratios of the 1G–TKIs were around 50-fold, meaning that this secondary mutation induced a strong resistance to 1G–TKIs (Table 1). Similar to the cells with L747S and D761Y mutations, the Ba/F3-L858R/T854A cell line exhibited enhanced sensitivities to 2G- or 3G–TKIs. In particular, the IC_50_ ratios of the 3G–TKIs in the Ba/F3-L858R/T854A cell line were similar to those in the Ba/F3-L858R cell line (IC_50_ ratios, around 1-fold) (Table 1). These findings suggest that irreversible EGFR-TKIs, especially 3G–TKIs, can overcome the resistance induced by uncommon secondary mutations. This tendency was also observed for the T790 M mutation (Table 1).

### Inhibitory activities of each generation of EGFR-TKIs for the phosphorylation of EGFR in cell lines with uncommon secondary *EGFR* mutations

**Fig. 4:**
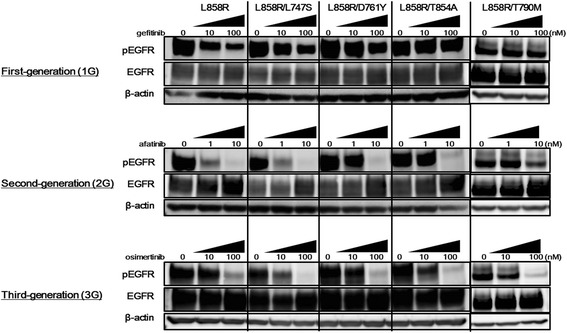
Western blotting for the EGFR signal. Western blotting was performed using each generation of EGFR-TKI (1G, gefitinib; 2G, afatinib; 3G, osimertinib). The samples were collected at 3 h after stimulation with each EGFR-TKI. When gefitinib was used to inhibit EGFR, the phosphorylation level of EGFR was significantly decreased in the Ba/F3-L858R cell line, compared with the other Ba/F3 cell lines harboring secondary mutations (L858R/L747S, L858R/D761Y, L858R/T854A and L858R/T790 M). In particular, the phosphorylation level of EGFR in the Ba/F3-L858R/T854A cell line was not reduced even by a high concentration of gefitinib (100 nM). Afatinib (2G) reduced the phosphorylation level of EGFR in the Ba/F3-L858R/L747S cell line to an extent similar to that observed in the Ba/F3-L858R cell line. Even in the Ba/F3-L858R/D761Y and Ba/F3-L858R/T854A cell lines, the phosphorylation of EGFR was inhibited by afatinib in a dose-dependent manner. Osimertinib (3G) reduced the phosphorylation level of EGFR in the Ba/F3 cell lines harboring secondary mutations (L858R/L747S, L858R/D761Y, and L858R/T854A) to an extent similar to that observed in the Ba/F3-L858R cell line. β-actin was used as an internal control

To investigate the differences in the EGFR inhibitory activities of EGFR-TKIs against cells carrying uncommon secondary *EGFR* mutations, western blotting was performed using each generation of EGFR-TKIs. We used gefitinib, afatinib, and osimertinib as 1G**-**, 2G**-**, and 3G–TKIs, respectively. When gefitinib was used to inhibit EGFR, the phosphorylation level of EGFR was significantly reduced in the Ba/F3-L858R cell line in a dose-dependent manner, compared with the phosphorylation levels in other Ba/F3 cell lines harboring an uncommon secondary mutation (Fig. 4). In particular, the phosphorylation level of EGFR in the Ba/F3-L858R/T854A cell line was not reduced even by a high concentration of gefitinib (100 nM). In contrast, irreversible EGFR-TKIs (2G, afatinib; 3G, osimertinib) reduced the phosphorylation level of EGFR in these Ba/F3 cell lines harboring an uncommon secondary mutation to a greater extent (Fig. 4). Especially, osimertinib (3G) reduced the phosphorylation level of EGFR in Ba/F3 cell lines harboring an uncommon secondary mutation to an extent similar to that seen in the Ba/F3-L858R cell line. This tendency is consistent with the difference in sensitivities; therefore, these findings suggest that the difference in sensitivities is caused by the difference in the EGFR inhibitory activities of each generation of EGFR-TKIs in the cell lines with uncommon secondary *EGFR* mutations.

## Discussion

The *EGFR* T790 M mutation in exon 20 is the most common secondary mutation and accounts for approximately 50%–60% of cases with acquired resistance to 1G–TKIs, while uncommon *EGFR* secondary mutations account for 1%–2% of resistant cases [20, 28, 29]. Since a large proportion of NSCLCs harbor *EGFR* mutations, especially among Asian patients, uncommon secondary mutations should not be ignored despite their relative scarcity. In the present study, we found that Ba/F3 cell lines harboring an uncommon secondary mutation (L858R/L747S, L858R/D761Y, or L858R/T854A), which are associated with resistance to 1G–TKIs, were sensitive to irreversible EGFR-TKIs, especially 3G–TKIs, suggesting that treatment with 3G–TKIs might be effective for the treatment of lesions with uncommon secondary *EGFR* mutations. Although the efficacy of 2G–TKIs against lesions with uncommon secondary mutations has been demonstrated previously [11–13], to the best of our knowledge, the present study is the first to investigate the in vitro sensitivities of cells with these uncommon secondary mutations to various EGFR-TKIs, demonstrating the efficacy of irreversible EGFR-TKIs, especially 3G–TKIs.

The most common *EGFR* mutations, exon 19 deletion and L858R, have both an increased affinity for reversible EGFR-TKIs and a decreased affinity for ATP, compared with wild-type EGFR [41, 42]. The common secondary *EGFR* T790 M mutation only modestly affects the binding of reversible EGFR-TKIs. More importantly, however, it restores the affinity for ATP, similar to that of wild-type *EGFR* [43]. The L747S mutation occurs at the start of the loop between the h3 strand and the α-C-helix, and the D761Y mutation is predicted to occur in the α-C-helix of EGFR [11, 12, 44]. These residues are adjacent to K745 and E762, which form a salt bridge that interacts with a- and h-phosphates when ATP is present [44], and are also adjacent to reversible EGFR-TKI binding sites. T854 is located at the “bottom” of the ATP-binding site, on the C-lobe [44]. Notably, the side chain of T854 is within contact distance of erlotinib or gefitinib in the active structure [42, 44] and is within contact distance of lapatinib in the inactive structure [45]. Therefore, these secondary mutations are thought to influence the binding affinity to ATP or reversible EGFR-TKIs. In our present study, the T854A mutation led to a strong resistance to 1G–TKIs, whereas the L747S and D761Y mutations led to weak resistances. The inhibitory activities of 1G–TKIs for the phosphorylation of EGFR in cell lines with these mutations, especially L858R/T854A, were weakened compared with those in cells carrying only the sensitive L858R mutation. In contrast, irreversible EGFR-TKIs, especially 3G–TKIs, were effective against the transfectant Ba/F3 cell lines harboring these secondary mutations, and the inhibitory activities of irreversible EGFR-TKIs for the phosphorylation of EGFR in cells with these mutations were similar to that in cells with the sensitive L858R mutation alone. Although the detailed mechanism of resistance to 1G–TKIs in cells carrying these secondary mutations remains unclear, the use of irreversible EGFR-TKIs (especially 3G–TKIs) that can inhibit EGFR independently of ATP competition might be useful for overcoming these resistances, based on our experimental findings. These findings indicate that secondary mutations might influence the binding affinity to ATP or reversible EGFR-TKIs, consistent with speculations based on crystal structures.

The discovery that 4-anilinoquinazolines exhibit EGFR inhibitory activity led to the development of 1G–TKIs (Fig. 1) [46]. 2G–TKIs have been developed from 4-anilinoquinazoline and bear Michael acceptor groups in the form of a reactive acrylamide, which is capable of forming covalent adducts with C797 of the EGFR protein (Fig. 1) [46]. Therefore, 2G–TKIs have both reversible (ATP competitive) and irreversible (covalent binding to C797) inhibitory effects. In the present study, 2G–TKIs were more effective against cells with these secondary mutations than 1G–TKIs, but 2G–TKIs were less effective against cells with these secondary mutations than against those with the sensitive L858R mutation alone. Since the secondary mutations can influence the binding affinity to ATP or reversible EGFR-TKIs, the lower effectiveness of 2G–TKIs against cells with these secondary mutations, compared with those with the sensitive L858R mutation alone, can be explained by their ATP competitive inhibitory effects. In contrast to 2G–TKIs, 3G–TKIs mainly have an irreversible inhibitory effect (covalent binding to C797), explaining the similarity in sensitivity between cells with secondary mutations and those with the sensitive L858R mutation alone.

Our present study had several limitations. First, the structures of the mutated *EGFR* after exposure to EGFR-TKIs and the binding affinity to ATP or reversible EGFR-TKIs could not be analyzed, and the detailed mechanisms responsible for these differences in sensitivity remain unclear. Our results, however, did reveal that the resistances induced by secondary mutations can be overcome using irreversible EGFR-TKIs, especially 3G–TKIs, indicating that these secondary mutations can influence the binding affinity to ATP or reversible EGFR-TKIs. Second, to confirm our experimental findings, further clinical data regarding these uncommon secondary mutations is required. Although their frequencies were very low in our database analyses, the exact frequencies remain unknown because the COSMIC database includes cancer types other than NSCLC and most of the analyzed samples (both TCGA and COSMIC) had never been treated with EGFR-TKIs. In addition, the samples were typically analyzed using detection assays that cannot detect uncommon mutations. Therefore, the actual frequencies might be higher than those reported here. Along with the introduction of 3G–TKIs into clinical settings, re-biopsies of tissue to test for acquired resistance are likely to be performed more frequently [19, 32, 33]. These uncommon secondary mutations, however, cannot be detected by most of the detection assays that are presently in clinical use. Therefore, more comprehensive analyses, such as next-generation sequencing, should be introduced into clinical settings so that patients who do not have T790 M but should nevertheless be treated with 3G–TKIs are not missed.

## Conclusions

Our present study showed that irreversible EGFR-TKIs, especially 3G–TKIs, can overcome the resistance induced by uncommon secondary mutations (L747S, D761Y, and T854A). Switching to 3G–TKIs might be a promising treatment strategy for acquired resistance arising from uncommon secondary mutations. To confirm these findings, both basic research and clinical research are additionally needed.

## Abbreviations

1G–TKI: First-generation reversible EGFR-TKI
2G–TKI: Second-generation irreversible EGFR-TKI
3G–TKI: Third-generation irreversible EGFR-TKI
COSMIC: Catalogue of Somatic Mutations in Cancer
DMEM: Dulbecco’s Modified Eagle’s Medium
EGFR: Epidermal growth factor receptor
EGFR-TKI: EGFR tyrosine kinase inhibitor
FBS: Fetal bovine serum
IC_50_: 50% inhibitory concentration
IL-3: Interleukin-3
MTT: 3,4,5-dimethyl-2H-tetrazolium bromide
NSCLC: Non-small cell lung cancer
PBS: Phosphate-buffered saline
RPMI: Roswell Park Memorial Institute
SD: Standard deviation
TCGA: The Cancer Genome Atlas
